# Stress and cancer: The mechanisms of immune dysregulation and management

**DOI:** 10.3389/fimmu.2022.1032294

**Published:** 2022-10-05

**Authors:** Yixin Liu, Sheng Tian, Biao Ning, Tianhe Huang, Yi Li, Yongchang Wei

**Affiliations:** ^1^ Department of Radiation and Medical Oncology, Zhongnan Hospital of Wuhan University, Wuhan University, Wuhan, China; ^2^ Hubei Key Laboratory of Tumor Biological Behaviors, Zhongnan Hospital of Wuhan University, Wuhan University, Wuhan, China

**Keywords:** chronic stress, hypothalamic-pituitary-adrenal axis, tumor microenvironment, immunity, stress management

## Abstract

Advances in the understanding of psychoneuroimmunology in the past decade have emphasized the notion that stress and cancer are interlinked closely. Durable chronic stress accelerated tumorigenesis and progression, which is unfavorable for clinical outcomes of cancer patients. Available evidence has provided unprecedented knowledge about the role and mechanisms of chronic stress in carcinogenesis, the most well-known one is dysfunction of the hypothalamus-pituitary-adrenal (HPA) axis and the sympathetic nervous system (SNS). With abnormal activation of neuroendocrine system, stress-related hormones contribute to increased oncogenes expression, exacerbated chronic inflammation and impaired immunologic function. In addition, accumulating studies have demonstrated that diverse stress interventions including pharmacological approaches, physical exercises and psychological relaxation have been administered to assist in mental disorders reduction and life quality improvement in cancer patients. In this review, we systematically summarize the connection and mechanisms in the stress-immune-cancer axis identified by animal and clinical studies, as well as conclude the effectiveness and deficiencies of existing stress management strategies.

## Introduction

With the fast pace of living in today’s society, stress is becoming a common experience across the globe. Stress origins from various changes in social environment including interpersonal relationships, financial hardships and physiological diseases, leading to adaptive psychological and physiological changes (1–3). Depending on its duration and intensity, stress can be classified as acute (e.g., surgery, exercise, cold temperature) or chronic (e.g., social isolation, work stress, prolonged cancer treatment). Generally, acute stress can quickly mobilize adaptive response as a stimulant to maintain homeostasis, which is beneficial to the body. However, when stressors don’t subside for a long time, they will exert effects on the endocrine system and behavioral responses to participate in multiple diseases (4, 5). It is widely accepted that there are tight associations between stressful events and cancer. Chronic exposure to stressors promotes tumorigenesis and development. Meanwhile, some psychosomatic symptoms such as anxiety, fatigue, or depression during survivorship can also act as the sources of stress to exacerbate tumor progression (6–9).

The neuroendocrine system, the most comprehensively studied mediator that links stress and cancer, consists of the hypothalamus-pituitary-adrenal (HPA) axis and the sympathetic nervous system (SNS) (2). Stress induces the release of three major hormones: epinephrine, norepinephrine and glucocorticoid. These stress-related hormones activate various cytokines secretion and molecular signaling pathways to remodel the tumor microenvironment (TME) through binding to adrenergic receptors (ARs) and glucocorticoid receptors (GRs) (1, 9). For example, behavioral stress-induced epinephrine accelerated tumor growth through β2AR/PKA/DAB pathway in a prostate-specific myc transgenic murine model (10). Similarly, chronic stress promoted norepinephrine secretion which induced VDCC phosphorylation and IGF2 exocytosis in lung epithelial cells to facilitate malignant transformation (11). As for the glucocorticoid, social defeat elevated the expression of glucocorticoid-inducible factor Tsc22d3, resulting in increased plasma corticosterone concentrations and damaged DC function (12). Besides, higher expression levels of GRs and glucocorticoid regulation kinases predicted poorer survival in breast cancer (13, 14).

Immunosuppression is one of hallmarks of TME. Most immune cells express β-ARs and GRs, they are the bridges to stress-induced immune function damage (15). Mounting animal studies have demonstrated that chronic stress could suppress cancer-related immune responses through altering various immune cells. In clinical researches, the metastatic breast cancer patients with more depressive symptoms showed higher plasma cortisol concentrations and suppressed immunity (16). Additionally, social isolation was associated with higher norepinephrine levels, while social support elevated the percentage of NK-T cells in ovarian cancer patients (17, 18). All of these researches indicate that there is a tight link between stress and tumor immunity.

In this review, we present a comprehensive interpretation of stress responses at organism level, and the interaction between stress and tumor immune microenvironment. In addition, we discuss the limitations and application perspectives of current stress management strategies in cancer patients.

## Epidemiological association between stress and cancer

With the rapid pace of life, mental health of people is growingly concerned. Psychosomatic disturbance is more common among individuals diagnosed with cancer as compared to general population (19). For example, the prevalence of depression among cancer patients ranges from 15-30%, while the average rate of general population is about 3.3% (19). It is noteworthy that although limited number of studies and non-uniform diagnosis standards (self-assessment report, symptom detection, or person-to-person interview) cause results heterogeneity, the incidence of psychologic disorders in cancer patients has gradually increased (20).

Psychological stress level reaches the first peak at the time of cancer diagnosis and psychosomatic symptoms containing anxiety, fatigue, insomnia even depression subsequently develop over the next year (21, 22). In an epidemiological survey including 8,387 individuals, cancer patients had the highest depression hazard ratio following the initial two years compared to people diagnosed with other diseases such as diabetes, hypertension, or stroke (23). In addition, the vulnerable groups who experienced significant psychological trauma were more susceptible to developing depressive episodes after cancer diagnosis (24). Interestingly, mental symptoms may appear before cancer in some patients. For example, pancreatic cancer patients suffered fatigue, anorexia, or insomnia for several months before the cancer diagnosis (25). A similar finding was reported in breast cancer, depressive symptoms have been detected in approximately 30% of women before the cancer diagnosis (26). Likewise, both invasive and early-stage ovarian cancer patients presented fatigue and weight loss for 2-7 months before diagnosis (27). In a case-control pilot study, malignant primitive brain tumors (MPBT) risk was significantly linked to major life events over the past 5 years before diagnosis, which indicated that psychological stress might contribute to MPBT appearance (28). Summarily, these results highlight that repetitive stress enables a permissive environment for primary cancer initiation, while tumorigenesis mechanisms may drive mental disorders.

After initial cancer diagnosis, stress levels will decline as a result of adaptive adjustments but reach peaks again during treatment and recurrence (9, 29, 30). In the process of cancer development, stress and affective disorders may accelerate cancer growth and metastasis (31–33). It is now quite clear that the presence of comorbid depression influences most hallmarks of cancer mediated by specific mechanisms including apoptosis resistance, vascularization, epithelial-mesenchymal transition (EMT) and evasion of immune destruction (7, 34). In 2008, a comprehensive meta-analysis was performed to assess the longitudinal associations between psychosocial stress and cancer outcomes (6). The results of this study indicated stress-related variables including stress-prone personality, emotional distress and depression had an adverse effect on cancer incidence and survival, despite publication bias existed. Another meta-analysis conducted in 16 prospective cohort studies initiated from 1994 to 2008 found that psychological distress was related to greater cancer mortality, especially in prostate and colorectal cancer (35). Among women with breast cancer, social stress was a critical driver of high mortality (36). Chronic stress also affects central nervous system tumors. For instance, sixty-one percent of the long-term brain cancer survivors experienced elevated stress levels despite their stable disease and less cancer-related concern (37). Of note, brain tumor patients may be reported with higher stress levels than other cancer populations because of the presence of cognitive and neuropsychiatric sequelae.

In addition to psychological elements, many physical factors are also involved in stress-related-tumor development, which are mainly reflected in the course of anti-tumor treatment (9). During perioperative period, patients are more likely to undertake the shedding risk of tumor cells (38, 39). Specifically, this unfavorable physical condition is a result from elevated inflammation responses and stress level originating from tissue damage, wound pain, hypothermia, and specific anesthesia. Another crucial time frame is adjuvant treatment. Despite their powerful tumor killing capacity, adjuvant therapies are always accompanied with side effects such as nausea, diarrhea or insomnia, which are also important components of stressors (40–42). Furthermore, the application of some chemotherapy drugs, like cisplatin and paclitaxel, can activate the inflammation stress pathway and induce various pro-tumorigenic factors expression to facilitate tumor development (43).

Taken together, stress presents in all the stages of tumor. Chronic exposure to multiple stressors increases the susceptibility to cancers, and psychosomatic disorders during survivorship can also contribute to elevated mortality.

## The interaction of stress, neuroendocrine and immunity in cancer

### Regulation of stress-related hormones in cancer

**Figure 1:**
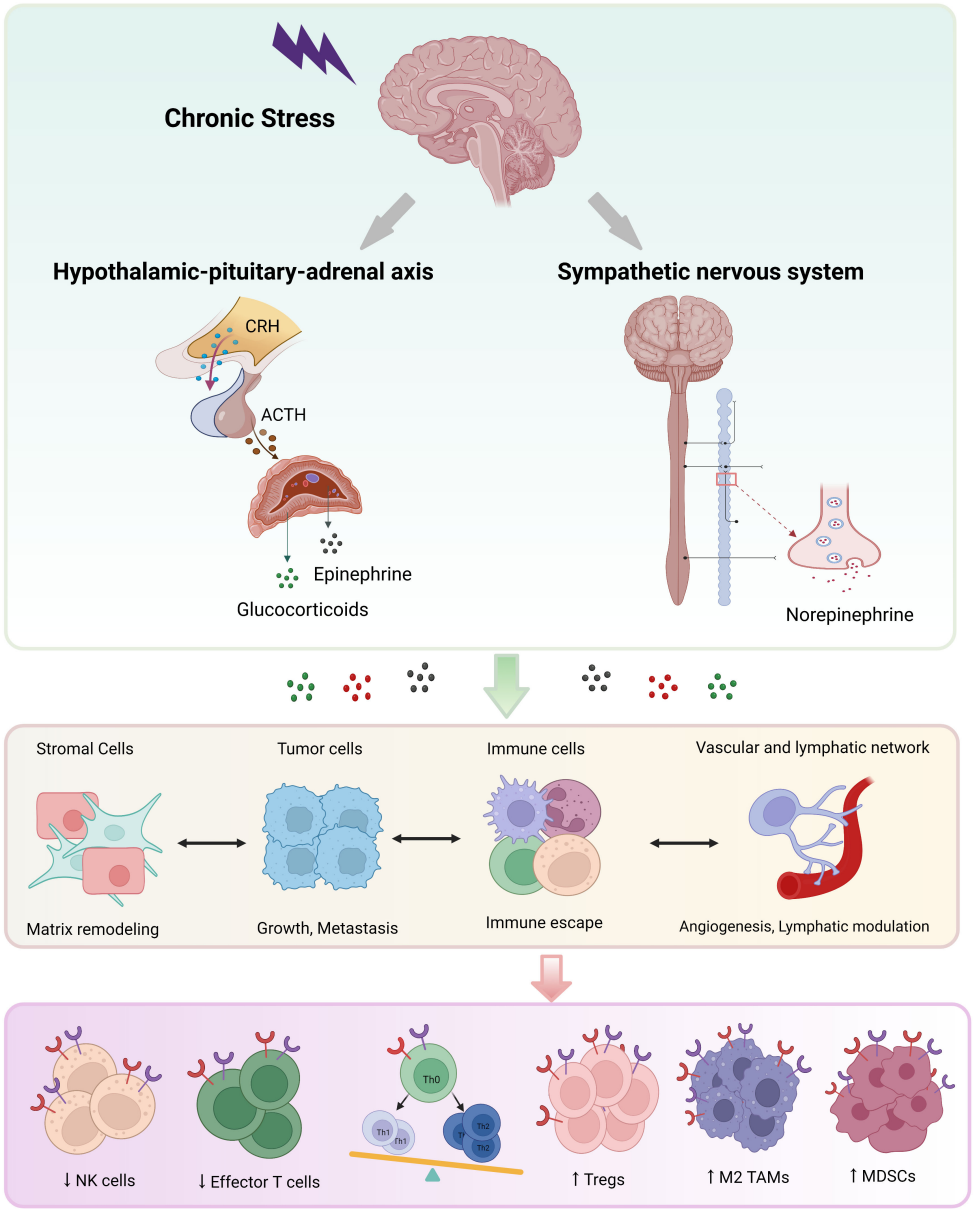
The neuroendocrine mechanisms of chronic stress. Chronic stress triggers activation of the hypothalamus-pituitary-adrenal axis and the sympathetic nervous system, leading to the secretion of catecholamine (mainly epinephrine and norepinephrine) and glucocorticoid. These stress-related hormones promote most hallmarks of cancer through binding to receptors on diverse cells, especially immune cells. NK, natural killer; DC, dendritic cell; Th, helper T; TAM, tumor-associated macrophage; MDSC, myeloid-derived suppressor cell; Treg, regulatory T cell.

The HPA axis and SNS are involved in stress-activated neuroendocrine system (1, 9). Chronic stress stimulates corticotropin-releasing hormone (CRH) release from the hypothalamus and adrenocorticotropic hormone (ACTH) secretion from the pituitary gland, then ACTH triggers the adrenal gland cortex to release cortisol into blood. Catecholamine (epinephrine, norepinephrine and dopamine) is produced from both the adrenal gland medulla and nerve fiber endings (4, 8, 44). All of these stress-related hormones play biological roles through binding to corresponding receptors distributed on the surface of diverse types of cells (**Figure 1**).

Previous studies have revealed that catecholamine and glucocorticoid promoted tumor initiation and metastasis, which were related to poor survival (31, 45, 46). Consistently, the activation of ARs and GRs signaling pathways could accelerate cancer progression under stress conditions (2, 9, 47). Indeed, the tumor-promoting effects of catecholamine are primarily mediated by the β2 adrenergic receptor (ADRB2) (48, 49). In breast cancer, chronic stress-induced epinephrine release enhanced LDHA-dependent glycolysis to promote stem-like properties of cancer cells (50). Additionally, adrenergic receptor β2 activation by stress-induced epinephrine could promote breast cancer progression through M2 polarization in tumor microenvironment (51). In prostate cancer mice model, researchers revealed that epinephrine activated the ADRB2/PKA/BAD pathway to accelerate cancer development and inhibit chemotherapy-related apoptosis under behavioral stress conditions (10). Similar findings were reported in gastric cancer, exposure to epinephrine altered neuroendocrine phenotypes of tumor cells, leading to invasion and metastasis *via* the β2-AR/MACC1/axis (31).

Norepinephrine is mainly produced from the adrenal gland cortex, and partly comes from nerve fiber endings. Several *in vivo* animal studies have assessed its effects on tumor progression. For example, chronic stress elevated norepinephrine levels, which then activated β2-adrenergic receptors and significantly inhibited the PPARγ expression to promote VEGF/FGF2-mediated breast cancer angiogenesis (52). Besides, stress-induced catecholamine activation facilitated M2 macrophage differentiation and induced a metastasis switch in the primary breast cancer mouse model (53). In ovarian carcinoma patients, social isolation promoted norepinephrine secretion, which was correlated with higher malignancies (46). The animal study also suggested that chronic stress stimulated norepinephrine release, resulting in FosB and IL-8-driven ovarian cancer growth (54). Moreover, norepinephrine contributed to the malignant transformation of normal tissues. After chronic unpredictable stress treatment, norepinephrine increased VDCC phosphorylation through ADRB/PKA pathway to facilitate lung epithelial cell malignant transformation and lung tumorigenesis (11).

Glucocorticoid (GC), an extremely critical regulatory molecule in the body, plays roles in growth, development, metabolism and immune regulation (55). In a rodent model, pretreatment with the anxiolytic drug chlordiazepoxide prevented the suppressive effects of psychological stress on innate IFN-gamma production through attenuating the stress-induced corticosterone concentrations (56). In addition, endogenous glucocorticoid assisted the transformation of immunosuppressive phenotype (PD-1+, TIM-3+, Lag3+) in CD8+ T cells (57). In hepatocellular carcinoma, high levels of GC during depression promoted progression by PD-1 upregulation in tumor-infiltrating natural killer (NK) cells (58). Social defeat elevated plasma corticosterone, as well as induced the expression of glucocorticoid-inducible factor Tsc22d3, which blocked dendritic cells (DCs) and T cell activation to weaken the immunotherapy (12). In addition to immune regulation function, chronic restraint-related glucocorticoid concentration could attenuate p53 function and promote tumorigenesis of colorectal cancer and breast cancer (59).

There are also several hormones participating in stress-mediated changes besides catecholamine and glucocorticoid. As a precursor of epinephrine and norepinephrine, dopamine inhibits tumor growth through the activation of different receptors (60, 61). For example, dopamine treatment blocked stress-induced angiogenesis and tumor growth in ovarian cancer (60). Likewise, chronic stress-mediated vascular growth in ovarian cancer models was prevented by activating the pericyte DR1/cAMP/PKA signaling pathway. Oxytocin (OXT) is a neurohormone released from the hypothalamus (61). Under chronic stress conditions, elevated oxytocin activated the ERK-VEGF/MMP-2 axis to promote lung metastasis of melanoma (62). Besides, Frick et al. (63) found that thyroid hormone levels were reduced under chronic stress, and lymphoma growth was altered. In a rat model, chronic stress could increase prolactin release to facilitate breast cancer, which was blocked by opioid receptor antagonist naltrexone (64).

The studies outlined in this section support that chronic stress alters neuroendocrine homeostasis to facilitate tumor progression, and some classic stress hormones can act as specific clinical biomarkers to evaluate cancer risk. Moreover, it is important to further explore more hormones involved in cancer stress, as well as investigate their mutual regulatory networks (65).

### Neuroendocrine regulation of tumor-associated immune cells

Given ARs and GRs are widely distributed on the surface of immune cells and sympathetic fibers innervate lymphoid organs and tissues, it is easy to conclude that there are interactions between neurons and immune system (49, 66, 67). According to the origin, tumor-associated immune cells are divided into myeloid and lymphocytes to participate in innate and adaptive immunity after maturity. In general, chronic stress suppresses immunogenic cell death embodied in impaired immune-supportive function and increased exhausted immune cells (34). These effects have been summarized in **Table 1**.

**Table 1:** Summary of stress-related immune cells regulation in cancer.

Cancer type	Stressor	Target	Effects	Ref
Breast Cancer	Operative stress	MDSCs	Metastasis	(68)
	Social isolation	TAMs	Tumor growth	(51)
	Cold stress	MDSCs	Tumor growth	(69)
	Cold stress	CTLs; Tregs	Immunotherapy failure	(70)
	Cold stress	CD8+ T cells	Metastasis	(71)
	Restraint stress	Macrophages	Metastasis	(53)
	Restraint stress Social Isolation	CD8+ T cells	Angiogenesis	(72)
Ovarian Cancer	Restraint stress	Macrophages	Tumor growth	(73)
	Restraint stress	Macrophages	Tumor growth	(74)
	Restraint stress	Macrophages	Metastasis	(75)
Colon Cancer	Social defeat	DCs; IFN-γ+ T cells	Immunotherapy failure	(12)
	Cold stress	CTLs	Tumor growth	(76)
	Scream sound stress	Th1; Th2	Tumor growth	(77)
	Wet cage exposure	NK cells	Treatment failure	(78)
Melanoma	Social disruption	DCs; CTLs	Immunotherapy failure	(79)
	Cold stress	CTLs	Tumor growth	(76)
	Wet cage exposure	NK cells	Treatment failure	(78)
Squamous cell carcinoma	Restraint stress	Tregs	Tumor growth	(80)
	Anxiety	CTLs; Tregs	Tumor growth	(81)
Lung cancer	Social defeat	DCs; IFNγ+ T cells	Immunotherapy failure	(12)
	Restraint stress	Macrophages	Lymph vasculature dissemination	(82)
Hepatocellular carcinoma	Restraint stress	Myeloid cells	Tumor growth	(83)
Prostate Cancer	Chronic unpredictable mild stress	TAMs	Tumor growth	(84)
Fibrosarcoma	Chronic subordinate colony housing	MDSCs; Tregs	Tumor growth	(85)
Lymphoma	Restraint stress	CD4+T cells	Tumor growth	(63)

As the first step in adaptive immune priming, DCs play a critical role in antigen presentation (86). Adrenergic stimulation of DCs suppressed their migration through β2-ARs-mediated modulation of interleukin production (87). Similarly, dexamethasone prevented DCs from undergoing maturation and priming T cells efficiently (88). In breast cancer, cold stress influenced phenotypes of DCs in both naïve and tumor-bearing mice (89). After exposure to social disruption, the full maturation and antigen uptake capacity of migratory and lymphoid-resident DCs were impaired, resulting in immunotherapy failure in a mouse model of melanoma (79).

NK cells are essential for innate immune surveillance and subsequent host defense against virus infection or cancer cells (90). NK cell function could be destroyed under different stress conditions. For instance, acute swim stress and epinephrine administration suppressed NK cell activity in rats (91). In hepatocellular carcinoma mice, depression upregulated the PD-1 expression in tumor-infiltrating NK cells and weakened their cytotoxicity (58). Similarly, surgery stress markedly decreased natural killer cytotoxicity (NKCC) through limiting FasL and CD11a expression (92). In breast cancer patients, higher stress levels were associated with decreased IFN-γ production and impaired NKCC (93, 94).

T lymphocytes mediate adaptive immune response and serve as a defender in anti-tumor killing immunity (95). Chronic stress alters the numbers of immune-supportive T cells (CD8+T) and immunosuppressive T cells (Treg), as well as the distribution of helper T (Th) cells-originated cytokines (15). Endogenous glucocorticoid originating from tumor monocyte-macrophage lineage cells could induce exhausted CD8+ T cell phenotype (Tim3+, PD1+) to reduce immune checkpoint blockade efficacy (57). In an anti-cervical cancer mouse model, propranolol strongly improved the STxBE7 vaccine efficacy by enhancing the frequency of CD8+ T infiltrating lymphocytes (TILs) (96). Additionally, chronic stress-activated β-AR signaling in tumor-bearing mice impaired the metabolism and function of TILs (76). As mentioned above, DC immaturity also affected subsequent recognition of tumor-associated antigens in cytotoxic T lymphocytes.

Other types of immune cells could also be influenced by chronic stress. For example, β2AR activation in CD4+ Foxp3+ T cells leaded to cAMP/PKA-dependent CREB phosphorylation and enhanced suppressive activity of Treg cells (97). In fibrosarcoma-bearing mice, psychosocial stress accelerated tumor growth through clustering Treg cells and myeloid-derived suppressor cells (MDSCs) in tumor tissues (85). Correspondingly, high-anxious mice showed faster UVB-induced squamous cell carcinoma growth accompanied by increased Treg cells and lower tumor infiltrating cytotoxic lymphocytes (CTLs) infiltration (81). Tumor-associated macrophages (TAMs) are also important components of TME. Prostate cancer patients with depression showed higher scores of CD68+ TAMs infiltration. And in mouse model, chronic stress predominantly promoted MDSCs mobilization and TAMs infiltration to facilitate tumor growth (84). Interestingly, the analysis of tumor specimens from socially isolated breast cancer patients indicated upregulated epithelial-mesenchymal transition gene expression profiles and elevated M2 polarization (98).

In this section, we establish a comprehensive understanding of the interaction between stress and tumor-related immune cells, which is likely to drive novel insights into anti-immunotherapy administration. However, how chronic stress influences other immune cells (e.g., B lymphocytes, cancer-associated fibroblasts), as well as their communication mode in stressful TME remains to be explored.

## The mechanisms in the stress-immune-cancer axis

**Figure 2:**
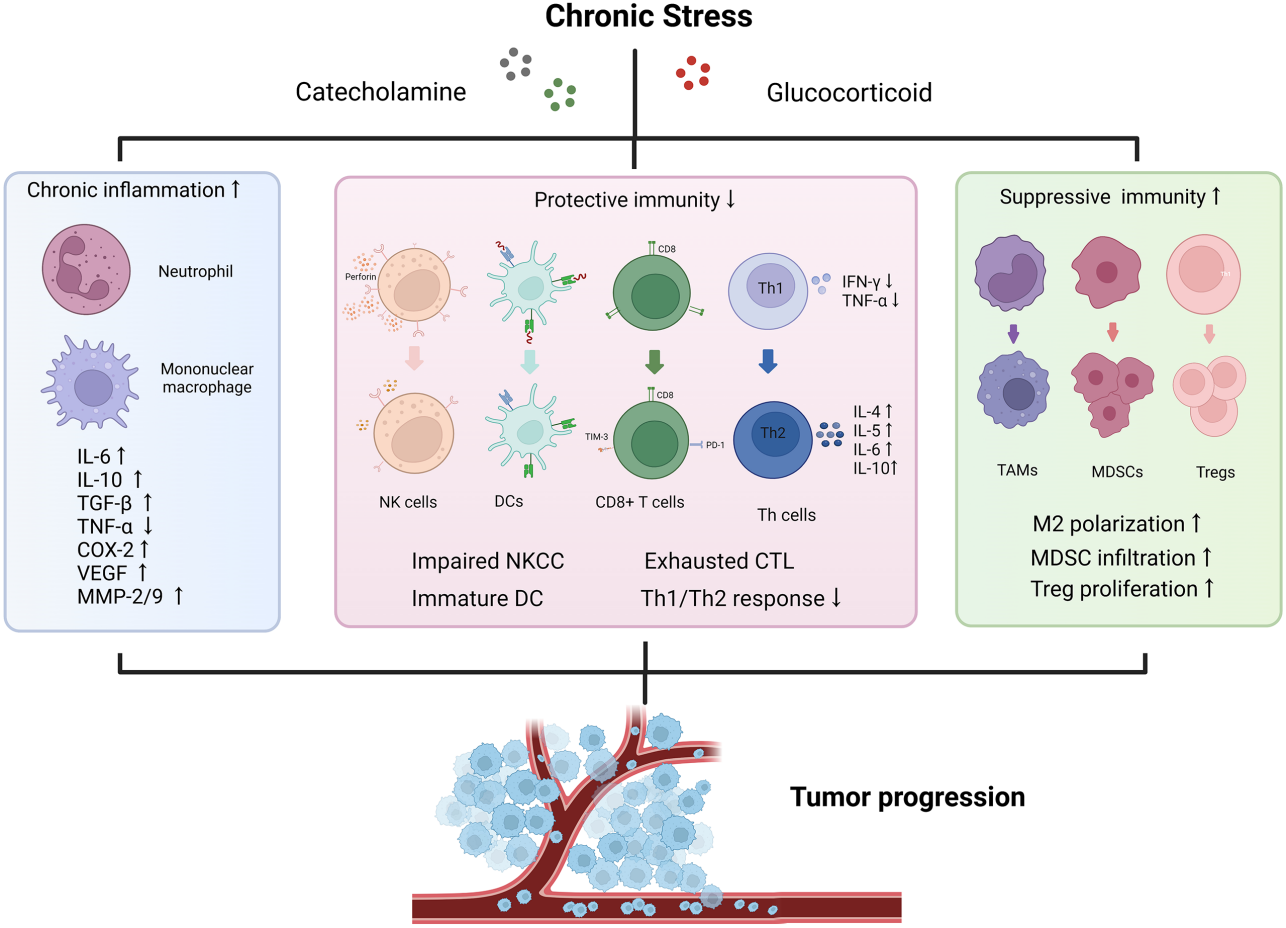
Effects of chronic stress on tumor immune microenvironment. The stressed tumor immune microenvironment is characterized with activated inflammation responses, impaired protective immunity and increased proportion of immunosuppressive cells. Chronic stress promotes the release of various pro-inflammatory and pro-tumor growth factors to accelerate tumor progression. IFN, interferon; TGF, transforming growth factor; TNF, tumor necrosis factor; VEGF, vascular endothelial growth factor; COX-2, cyclooxygenase-2; MMP, matrix metalloproteinase; NKCC, natural killer cell cytotoxicity; DC, dendritic cell; CTL, cytotoxic lymphocyte; Th, helper T cell; TAM, tumor-associated macrophage; MDSC, myeloid-derived suppressor cell; Treg, regulatory T cell.

The potential mechanisms of stress induced immunosuppressive TME have been widely investigated (34, 66). Long-term exposure to stressors leads to the concentration of classical catecholamine and glucocorticoid, which are recognized by receptors distributed on immune cells’ surface to reshape the immune microenvironment, and ultimately, drive tumor initiation and progression. Immune responses are divided into three functional categories: inflammation, immune protection, and immune suppression. Here we perform an integrative interpretation and discussion about immune dysregulation mechanisms from these three aspects (**Figure 2**).

### Inflammatory activation in cancer

Inflammatory immune responses (IIRs) are adaptive reaction of immune cells to changes in internal and external environment. Moderate IIRs protect the body from pathological damage, while overactivation causes damage (99). It is well known that inflammation contributes to tumor development through oxidative stress damage, DNA mutation and cytokines release. Moreover, there are some tumor initiation-related chronic inflammations such as ulcerative colitis and chronic gastritis, suggesting the strong link between inflammation and cancer (100, 101).

Chronic stress induces sustained elevation in the NF-κB signaling pathway and circulating pro-inflammatory factors (e.g., IL-1β, IL-6, IL-8, TNF-α) to contribute to immune disorders. As we previously reported, gastric adenocarcinoma patients with depression exhibited upregulated oxidative stress states and inflammation responses. In animal model, reactive oxygen species (ROS) was involved in the development of gastric cancer-related depression through inflammation (102). Likewise, stress-induced sympathetic nervous activation promoted tumor invasion *via* increasing pro-tumor growth cytokines (e.g., MMP-2, MMP-9, VEGF) (103). Studies documented higher peripheral levels of pro-inflammatory cytokines in mental disorder patients than healthy controls (104, 105). Within 4-8 weeks after surgical resection, the breast cancer patients with depressive symptoms showed greater serum TNF-α, IL-1β, and IL-6 levels (106). Correspondingly, higher IL-6 levels in breast cancer patients were associated with major depressive disorder (107). The whole genome transcriptional profiling identified depressive symptoms had potential links to dysregulation inflammatory biology in metastatic renal cell carcinoma (108). In an animal study, stress elevated the expression of inflammatory cyclooxygenase-2 (COX2), which was required for following lymphatic remodel and tumor dissemination mediated by VEGFC (82). Similar results were remarkably revealed in patients with ovarian cancer. The patients who lacked social support and experienced worse depression showed higher MMP-9 expression in TAMs (109). Besides, exosome analysis of ovarian cancer patients with lower social support exhibited activated inflammation and mesenchymal polarization (110).

Chronic stress also contributes to cancer-related psychiatric symptoms (19). The pro-inflammatory cytokines produced in the periphery can permeate the blood-brain barrier to impair cognitive processing. For instance, repeated social defeat significantly increased the trafficking of macrophages to brain and inflammatory markers expression on microglia, which leaded to anxiety-like behavior (111). Thus, the cross-talk along CNS and systematic inflammation is largely responsible for anxiety-like and depression-like behaviors, meanwhile, contributes to carcinogenesis (112).

### Imbalance of protective and suppressive immune responses in cancer

Tumorigenesis is characterized by immune escape, and immuno­surveillance is essential for effective antitumor therapies. Protective immunity is a rapid-activated response to eliminate infections, eradicate tumor cells and intensify vaccine effectiveness including both innate and adaptive immune (66, 113). The alterations in epigenetics and growth dynamics of tumor cells lead to the extracellular secretion of various cytokines that recruit suppressive immune cells (Tregs, MDSCs, TAMs) which are in favor of tumor growth (114).

Chronic stress inhibits protective immunity (113). For example, norepinephrine treatment elevated ADRB2 expression and inflammatory chemokines release in memory CD8+T cells but reduced their proliferation (115). Besides, corticosteroids inhibited DCs maturation leading to blocked Th1 immune responses (88). In clinical studies, the early-stage breast cancer patients with depression produced more Th1 cytokines (IL-12 and IFN-γ), while lower Th1 cytokines (IL-2) and NKCC (57, 97). Similarly, physiologic stress after surgical resection inhibited NKCC and T-cell responses of breast cancer patients (116). In patients with ovarian cancer, depression suppressed the cytotoxicity of NK cells and TILs, while social support alleviated these negative effects (17, 18, 117). More importantly, numerous animal models have been established to observe the effects of stress on tumor immunity. Chronic scream exposure induced freezing behavior in mice and caused a shift from the Th1 to the Th2 responses which significantly promoted cancer progression (77). When exposed to UV radiation, stressed mice had a shorter time to form skin tumors and showed lower IFN-γ, CCL27/CTACK expression levels and fewer CD4+ infiltrating cells than control group (80, 81). In terms of distant metastasis, the operative stress increased MDSCs infiltration in the breast cancer microenvironment to assist the EMT process through TGF-β1, VEGF, and IL-10 secretion, finally leading to pulmonary metastases (68). Additionally, chronic unpredictable stress markedly decorated a pre-metastatic niche through increasing the infiltration of macrophages into lung to promote metastatic colonization by tumor cells (32).

The distributions and functions of immune cells are essential for the success of anti-cancer immunotherapy (118). Any therapeutic strategies based on the immune system can be impaired by chronic stress. For instance, social disruption stress prevented DCs from undergoing full maturation upon antigen uptake and priming CD8+ T cells-mediated cytotoxicity, which finally impaired the effectiveness of vaccines in a murine melanoma model (79). A recent study suggested stressed-induced Tsc22d3 expression is a glucocorticoid-inducible factor, which was sufficient to impair DCs function and IFN-γ+ T cell activation, ultimately weakened anti-PD-1 therapy in fibrosarcoma mice (12).

### Other potential biological mechanisms

Detecting the mechanisms of stress-induced immunity change in cancer is becoming a hot topic (66, 113). As important responding organs to deliver multiply signals derived from internal and external environment, the brain and gastrointestinal (GI) tract are connected by a bidirectional signaling network called gut-brain axis (119–121). The intestinal microbiota is a critical regulator during gut-brain communication process, and participate in psychological disorders. For example, the patients with major depressive disorders had increased levels of Enterobacteriaceae and Alistipes but reduced levels of Faecalibacterium when compared to healthy people (122). Chronic stress can influence microbiota distribution and increase gut permeability. A recent study reported that rats which were more vulnerable to social defeat showed increased expression of immune-modulating microbiota such as Clostridia. And when naive rats were treated with microbiota from depression rats, they showed higher microglial density and IL-1β expression (123). In another animal research, chronic restraint stress affected Firmicutes/Bacteroidetes ratio through Inflammasome-mediated caspase-1 activation to accelerate anxiety symptoms and depressive-like behaviors (124). There are also connections between intestinal microbiota and cancer immunity (125, 126). Enterotoxigenic Bacteroides fragilis (ETBF) promoted tumorigenesis through an IL-17-driven MDSCs generation (127). In colon cancer patients, Fusobacterium nucleatum positivity was significantly associated with microsatellite instability (MSI)-high status and impaired adaptive immunity mediated by T cells (128). Furthermore, altering commensal gut bacteria has been implicated to weaken the effects of tumor immunotherapy (125, 129, 130). In light of this, several researches have been implied to explore the role of gut microbiome in stress-related tumor progression. Cui et al. (131) reported chronic restrained treatment altered mice gut microbiome composition and promoted breast cancer development. However, this field is in its nascency, and specific mechanisms of gut dysbiosis-related tumor progression in stress condition remain unclear.

In addition, inflammatory cytokines led to neuroendocrine cycle dysregulation and severe sleep disorders which were related to poor clinical outcomes in metastatic colorectal cancer and breast cancer patients (2, 19, 132). Metabolic stability is the basis of physiological functions in all organisms. Since immune activation is also an energy-consuming process, it is feasible to explore the relationship between metabolism and immunity under stress conditions. A recent study has indicated physical stress-induced leukotriene B4 triggered severe purine metabolic disorders in CD4+ T cells, which further leaded to a variety of anxiety-like behaviors in mice (133).

## Stress management and anti-cancer treatments

A significant proportion of cancer patients are burdened with varying degrees of chronic stress and this is likely to exert deleterious effects on clinical prognosis (7). It has been reported that appropriate stress modification was efficient to psychological adaptation and favorable clinical outcomes, which provided feasibility to combining stress management and anti-cancer approaches (2, 9).

### Blockages of stress-induced hormones

Hormones blockage is an efficient stress manage strategy. Researchers have confirmed the anti-tumor effects of adrenergic receptor antagonists, especially β-ARs antagonists (7, 134, 135). For example, propranolol treatment and β2-AR knockout decreased exhausted T cells infiltration and inhibited melanoma growth in mice (76). Low-dose β-ARs blocker (propranolol) therapy reduced MDSCs accumulation and relieved thermal stress-induced breast cancer growth (69). Besides, propranolol enhanced the sensitivity of gastric cancer cells to radiation through the NF-κB-VEGF/EGFR/COX-2 pathway (136). Ablation of the sympathetic nerve and β-adrenergic signaling blockage significantly inhibited stress-induced lung colonization of tumor cells (32). Furthermore, combined administration of propranolol and chemotherapy could improve the life quality of epithelial ovarian cancer patients (137). In a nested case-control study containing 4113 pancreatic patients, the individuals who used selective β1-blockers or non-selective β-blockers had lower cancer risk (138). ICI118,551, a specific ADRB2 antagonist, suppressed gastric cancer metastasis *via* inhibiting the ERK1/2-JNK-MAPK pathway (139). Similar results were also demonstrated in hepatic and pancreatic cancer (33, 140).

In addition to common adrenergic inhibitors, many antioxidants also showed anti-tumor activity. As but one example, melatonin remarkedly reduced the abdominal metastasis of ovarian cancer induced by chronic restraint stress, and this impact was partially mediated by the inhibition of the NE/AKT/β-catenin/SLUG axis (75). Considering the role of stress in immune regulation, hormone blockers may be ideal partners for immunotherapy. For instance, propranolol strongly increased the density and PD-1 expression of CTLs, which promoted the anti-tumor toxicity of STxBE7 vaccine in rodent models (96). Similarly, mifepristone administration reversed the negative impact of stress, as well as facilitated prophylactic immunotherapy effectiveness in socially defeated mice (56).

Summarily, the beneficial effects of propranolol have been demonstrated in many clinical researches (3, 137, 141, 142), and there are also two randomized controlled trials (RCTs) that initially combined propranolol and etodolac (COX-2 inhibitor) in breast and colorectal cancer (143, 144). All of these clinical findings have proved the application of β-ARs antagonists was a promising strategy to manage stress in cancer patients.

### Psychosomatic behavior management

Psychosomatic symptoms intervention suggests a novel therapeutic strategy for cancer patients experiencing chronic stress (66). Cognitive behavior treatment (CBT) is a kind of psychotherapy approach aimed to restructure patients’ cognition. The non-metastatic breast cancer patients who participated in cognitive intervention had a well-maintained decreased cortisol levels and improved relaxation ability (109). In another RCT of breast cancer, women in the CBT-based stress management group showed decreased emotional distress, upregulation in cellular immunity and better health condition in comparison with the control group (145, 146). In addition, previous studies revealed that mindfulness-based approaches exhibited strong capacity in relieving cancer-related stress (147). For example, mindfulness intervention provided metastatic cancer patients with an opportunity for pain control and spiritual relief, which possibly reinforced the effectiveness of early palliative care (148). Moreover, the brain’s reward system activation attenuated SNS-mediated MDSCs development in bone marrow, followed by less immunosuppressive and tumor growth inhibition in a murine model (149).

Considering the psychological damage in cancer patients, researchers have found that enriched living environment and social support were helpful to improve overall life quality (2). For example, when mice were housed in an enriched environment, they displayed enhanced NK cell immunity and retardative tumor malignancies (150). Besides, the enriched environment activated peripheral SNS/β-ARs/CCL2 signaling pathway, which protected mice from liver neoplasia growth and PD-L1 treatment resistance through increasing CD8+ T cells cytotoxic effect (151). In a study about community life support programs, the cancer patients experienced better quality of life after volunteer-provided palliative (152). The potential of dietary strategies in influencing carcinogenesis has been well established in epidemiological researches (19). Mediterranean diet, characterized by a higher intake proportion of monounsaturated, fruits and vegetable intake, is related to plasma stress-induced cortisol expression in adolescents (153). Furthermore, fatigue reduction diet for breast cancer patients contributes to improvements in sleep quality and fatigue symptoms (154).

Since moderate physical exercise plays a vital role in modulating neuroendocrine activity and strengthening immunosurveillance, researchers have tried to explore the influences of exercise on psychosomatic function in cancer survivors. Compared to control group, the individuals with hematological cancers who underwent an adjunctive exercise program during treatment were reported with less physical fatigue and greater physical function (155). Similar results were indicated in solid tumors, a 12-week exercise program could reduce anxiety and depression in lung cancer and prostate cancer patients (156, 157). Moreover, as a kind of new-style sport, yoga was an efficacious way to relieve cancer-related depression and improve sleep disturbance among cancer survivors (158–160). Additionally, other physical stress interventions including massage, Tai-Chi, and Chinese acupuncture have been also tested in cancer treatment (2, 66).

Although psychosomatic interventions have shown promising results in anti-cancer treatment, there was still insuficient evidence to support its clinical application. In a comprehensive review, the authors summarized 22 RCTs about psychosocial stress-reducing strategies, and they pointed out that most intervention did not significantly improve long-term cancer outcomes. Additionally, some of these studies had been criticized for methodological deficiencies (9). Hence, the administration of psychosomatic management strategies in cancer treatment should be more carefully explored.

### Anti-depressants application

The idea of anti-depressants application in cancer treatment is highly controversial. In animal studies, fluoxetine treatment after melanoma cells injection inhibited tumor growth, whereas chronic pre-treatment with fluoxetine before melanoma bearing accelerated metastasis formation through impairing protective immune responses (161, 162). Inconsistent results were also found in clinical practice (163, 164). In a double-blinded trial, fluoxetine treatment was well tolerated in advanced cancer patients and showed prevalent effectiveness in anxiety and depression reduction (165). Some anti-depressant drugs (e.g., paroxetine) could alleviate the side effects of chemotherapy like nausea and anorexia (19). Nevertheless, long-term intake of tricyclic antidepressants (TCAs) and selective serotonin reuptake inhibitors (SSRIs) might increase the risk of breast and ovarian cancer (166, 167). In a RCT enrolled 189 patients with advanced cancer, sertraline did not exert significant improvement in symptoms and survivorship (168). Collectively, the effects of anti-depressants on cancer populations are largely unclear and more researches are needed to fully evaluate their impact on long-term outcomes.

## Conclusion and perspectives

It is widely accepted that stress exposure creates negative influences on most hallmarks of cancer to facilitate tumorigenesis and progression. Meanwhile, cancer patients are vulnerable to psychosomatic disorders (e.g., insomnia, anxiety and depression), which are also major sources of chronic stress (7, 34, 66). The underlying molecular mechanisms have been identified in numerous pre-clinical studies, and we concluded the interaction of stress, neuroendocrine and cancer immune systems in this review. In general, chronic stress exerts positive effects on the HPA axis and SNS activation, which finally results in inflammation upregulation and suppressive cellular immunity toxicity. Under the mediation of multiple cytokines, immune cells communicate with circulating cancer cells in TME to promote tumor growth, angiogenesis and metastasis.

Given the close link between stress and cancer, the importance and effectiveness of stress management have been stated (2, 9). However, there are still some technological controversies in animal models and methodological deficiencies in clinical trials, as discussed below. 1) Although most preclinical studies show satisfactory results, some studies reach unexpected conclusions. For example, in a social isolation mice model, chronic stress suppressed mammary tumor growth through β-Adrenergic signaling (169). These contrary results are partly attributed to the subtle discrepancy of different stressors exposure (e.g., restraint stress, cold stress and social defeat). Approximately 30-40 percent of mice showed a resilient phenotype after social defeat (170), which meant the duration and intensity of stress exposure were critical. The detection of pre-existing stress levels in mice before modeling is also a critical problem that we usually ignore (34, 171). In addition, it is necessary to construct appropriate animal models according to purposes. When the stressors exposure predates tumor injection, researchers focus on the cancer susceptibility in the individuals experienced chronic stress, while the opposite condition pays attention to comprehensive influence of stress on tumor development (34). Finally, exogenous catecholamine or glucocorticoid treatment is difficult to simulate neuroendocrine changes of the body under stress condition. 2) As we briefly reviewed in last section, clinical stress-reducing interventions yield mixed effects on long-term cancer outcomes. RCTs so far usually use different treatment protocols (e.g., initial time and duration) and these heterogeneities may be the source of inconsistent results (9). Another problem to be aware of is methodological deficiencies. For example, assignment bias might cause the false positive conclusion of psychological interventions (172, 173). Actually, only a few positive outcomes have been replicated for validity because of various practical difficulties in the implementation of clinical trials.

The discrepancy between preclinical and clinical outcomes suggests the following aspects need to be further clarified in future studies. The first question is “who can benefit”. Physical examination and psychological tests play a critical part in this step. For example, the population who are older or psychologically vulnerable may benefit from psychological interventions (174). Importantly, cancer patients with cardiovascular disease should be given more caution when using β-ARs blockages (7). In addition, most of current available clinical evidence focuses on breast cancer and it is still a problem to address understudied populations. The next one is “when to intervene”. The interventions during appropriate phases may bear great effects. Although it is quite difficult to synchronize the critical periods of cancer initiation with stress, perioperative and adjuvant treatment phases during cancer treatment are more sensitive to stress-reducing interventions (9). The last one is “how to intervene”. As growing types of stress management strategies show effects on relieving psychological symptoms, present questions move to how to combine these approaches to improve long-term clinical outcomes in cancer patients. It has been demonstrated that multiple stress management models simultaneously containing relaxation training, cognitive behavior treatment and health education showed promising treatment capacity (66). The internet-based remote management programs also effectively improve the quality of life of cancer patients (175). Of note, it is still an unexplored field about the combined application of immunotherapy and psychological interventions in cancer treatment.

In conclusion, we provide a wide insight into the multiple interactive biobehavioral molecular pathways linking stress, immunity and cancer. Despite the absence of unified stress measurement tools and specific efficacy evaluation criteria makes stress difficult to explore, personalized stress management based on different pharmacological and psychosocial approaches will become an important component of tumor precision therapy in the future.

## Author contributions

YW developed the concept and design. YLiu and ST wrote the manuscript. BN, TH, and YLi provided critical discussion and edited the manuscript. All authors listed have made a substantial contribution to the work and approved the final version of the manuscript.

## Funding

This study was funded by the Oncology Science and Technology Innovation Cultivation Program of Zhongnan Hospital of Wuhan University (NO.2020-B-06).

## Acknowledgments

The authors thank all the members of the group for the critical reading of the manuscript.

## Conflict of interest

The authors declare that the research was conducted in the absence of any commercial or financial relationships that could be construed as a potential conflict of interest.

## Publisher’s note

All claims expressed in this article are solely those of the authors and do not necessarily represent those of their affiliated organizations, or those of the publisher, the editors and the reviewers. Any product that may be evaluated in this article, or claim that may be made by its manufacturer, is not guaranteed or endorsed by the publisher.
